# Acute Respiratory Infection Unveiling CPT II Deficiency

**DOI:** 10.3390/ijms19102950

**Published:** 2018-09-27

**Authors:** Nicolas Blah, Bénédicte Sudrié-Arnaud, Stéphanie Torre, Stéphane Marret, Soumeya Bekri, Abdellah Tebani

**Affiliations:** 1 Department of Metabolic Biochemistry, Rouen University Hospital, 76000 Rouen, France; nicolas.blah@etu.univ-rouen.fr (N.B.); B.Sudrie-Arnaud@chu-rouen.fr (B.S.-A.); Soumeya.Bekri@chu-rouen.fr (S.B.); 2 Department of Internal Medicine, Rouen University Hospital, 76000 Rouen, France; 3 Department of Neonatal Pediatrics, Intensive Care and Neuropediatrics, Rouen University Hospital, 76000 Rouen, France; Stephanie.Torre@chu-rouen.fr (S.T.); Stephane.Marret@chu-rouen.fr (S.M.); 4 Normandie Université, UNIROUEN, CHU Rouen, INSERM U1245, 76000 Rouen, France

**Keywords:** carnitine palmitoyl transferase, CPT II, rhabdomyolysis, acute infection, energy failure, beta oxidation

## Abstract

Carnitine Palmitoyl transferase 2 (CPT II) is involved in long-chain fatty-acid mitochondrial transport. Three clinical phenotypes of CPT II deficiency have been described: Lethal neonatal onset, infantile severe form, and the late onset more common muscular form. The muscular form of CPT II deficiency is characterized by pain crises and rhabdomyolysis triggered by energy-dependent factors. This form has been described as a benign condition; however, the acute crises are insidious and thus, pose a risk of death. We report a 3-year-old female child with an acute pulmonary infection and a concomitant rhabdomyolysis. The acylcarnitine profile was consistent with CPT II deficiency and a molecular study allowed the identification of the common missense variant (NM_000098.2: c.338C>T – p. Ser113Leu) at the homozygous state. The striking difference between the initial cause and the decompensation severity prompted us to consider other diagnoses. Deciphering the symptoms linked to CPT II deficiency among those of the initial decompensation results in initiating a timely a targeted therapy.

## 1. Introduction

**Figure 1:**
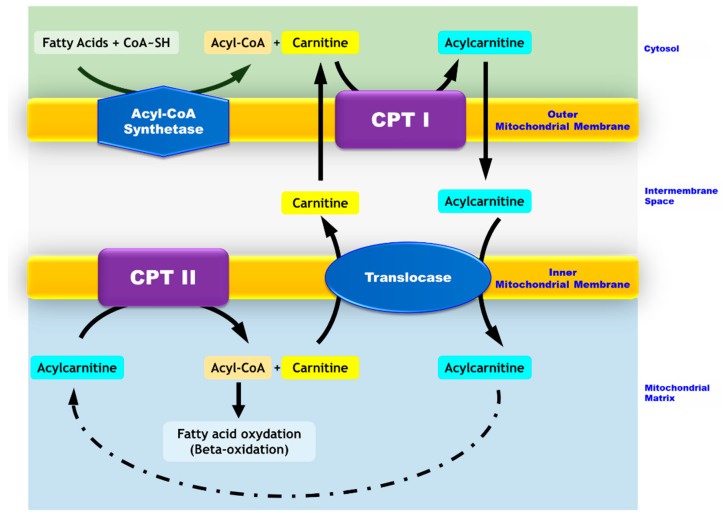
Mitochondrial transport of long-chain fatty acids. While medium- and short-chain fatty acids enter the mitochondria freely, long-chain fatty acids require carnitine-dependent transport to cross the mitochondrial membranes. In the cytosol, long chain fatty acids are converted to acyl-CoA by the acyl-CoA synthase. The carnitine palmitoyltransferase I (CPT I) located in the outer mitochondrial membrane catalyzes the conversion of acyl-CoA into acylcarnitines. The latter crosses the outer membrane and goes through the inner membrane via a transporter named translocase. Carnitine palmitoyltransferase II (CPT II), which is located in the inner mitochondrial membrane, allows for the conversion of acylcarnitines into acyl-CoAs and free carnitine. Carnitine exits the mitochondria via the translocase and thus, is recycled to form other acylcarnitines. Acyl-CoAs are directed toward the beta-oxidation process.

Carnitine Palmitoyl transferase 2 (CPT II) deficiency belongs to the mitochondrial beta-oxidation disorder family. This autosomal recessive disease is due to a defective transport of long-chain fatty acids from the inter-membrane space to the mitochondrial matrix where beta oxidation occurs (Figure 1). The clinical manifestations are related to a decreased beta-oxidation rate and the subsequent decrease in ATP concentration in the cells which rely on fatty acid oxidation for their energy production (muscle, heart) [1]. Three clinical phenotypes have been described: Lethal neonatal onset with multisystemic alterations [2], infantile severe form which affects mainly the Central Nervous System (CNS), liver, heart and muscles [3], and the late-onset mild muscular form [4]. Patients with the muscular form present with recurrent muscular pain episodes with rhabdomyolysis that can possibly lead to renal failure, respiratory insufficiency, and arrhythmia. Several mechanisms underlie the subsequent cell damages (i) the absence of ketone body production and (ii) the inhibition of Krebs-cycle enzymes by non-metabolized long chain fatty acids with a consecutive myocardial toxicity. Rhabdomyolysis results from skeletal muscle damage and the alteration of plasma membrane integrity with a subsequent release of the intracellular biomolecules into the bloodstream (CPK, myoglobin). The main pathophysiological mechanism underlying rhabdomyolysis is related to calcium homeostasis. Indeed, a decrease in ATP production and fragilization of the plasma membrane lead to an increased level of cellular free ionized calcium. This increase alters cellular functions, such as skeletal muscle cell contractility and mitochondrial metabolism and, ultimately, leads to cell death [5,6].

Muscular CPT II deficiency may remain asymptomatic and an acute crisis may be triggered upon an energy crisis, excessive energy expenditure, or a shortage of energy supply such as infection, prolonged exercise, or fasting. Muscular CPT II deficiency is often misdiagnosed and specific preventive and treatment managements are postponed. The diagnosis is delayed due to the unspecific clinical features, which may be masked by the clinical effects of the triggering factor such as infection. Besides, the biological diagnosis is tricky; an acylcarnitine profile is analyzed in routine practice and a CPT II deficiency acylcarnitine profile is informative with elevated concentrations of long-chain acylcarnitines, C16, C18, C18:1. However, this profile is often normal between the acute crises. CPT II enzyme activity assessment is reliable but a molecular study is usually conducted directly [1,4,7].

In this study, the case of a child with a CPT II deficiency is reported to emphasize that raising the awareness of intermittent presentations of CPT II deficiency is challenging.

## 2. Clinical History and Background

A 3-year-old female child was hospitalized in the pediatric department for dyspnea and fever (39.9 °C). She had a normal psychomotor development with no particular medical history. A week before, she was seen by a general practitioner for fever, bronchial congestion, odynophagia, cough and rhinitis. A chest X-ray showed a pulmonary right basal opacity and the patient was referred to pediatric emergency service. At presentation, she was polypneic and suffered from muscular pain with a fast worsening of the general state. RSV (Respiratory Syncitial Virus) type B has been identified as the cause of the respiratory infection. Besides, biological investigations showed a major inflammatory syndrome (C-Reactive Protein: 94 mg/L—N < 5) an elevation of transaminases (ASAT: 3412 UI/L, ALAT: 1210 UI/L—N: 10–35) with a major increase of Creatine PhosphoKinase (96,000 UI/L N: 50–170) and were consistent with an acute rhabdomyolysis. Further metabolic evaluations included urinary organic acid chromatography, a blood acylcarnitine profile, ketone bodies and lactate/pyruvate assessments.

**Figure 2:**
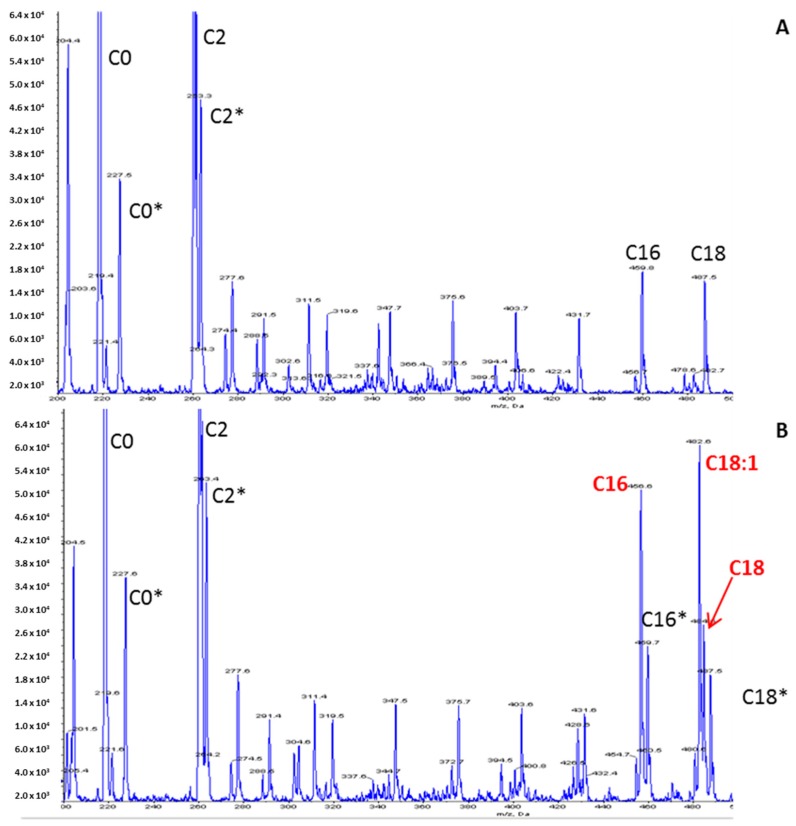
Acylcarnitine profile using an LC-MS/MS method. (**A**) Control; (**B**) Patient. Peaks represent butylated esters of acylcarnitines acquired by precursor ion scanning of 85 *m*/*z* in positive ion mode. Derivatized samples were injected into a Sciex 4000 QTRAP mass spectrometer (Sciex, Framingham, MA, USA) using an autosampler. Peaks corresponding to isotopically labeled internal standards are denoted by an asterisk.

The increased long-chain acylcarnitine concentrations (C16: 1.67 µmol/L—N < 0.27; C18: 1.11 µmol/L—N < 0.09; C18:1: 2.13 µmol/L—N < 0.42), the elevation of the calculated (C16:0 + C18:1)/C2 ratio 0.184 (N: 0.011–0.048) and the decrease in the free carnitine concentration (26 µmol/L—N > 30) on the acylcarnitine profile (Figure 2) were suggestive of CPT II deficiency. The other metabolic investigations were normal. Raising the hypothesis of CPTII deficiency helped to manage the acute phase by considering lipid avoidance and glucose infusion. The hospitalization was marked by the progressive regression of dyspnea, muscular pain and partial regression of rhabdomyolysis biological features.

Secondarily, a molecular study of the *CPT II* gene allowed for the identification of the common missense variant (NM_000098.2: c.338C>T – p. Ser113Leu) in the homozygous state, which is reported as a frequent variant in the muscular form of CPT II deficiency. This variant was found in a heterozygous state in both the unaffected parents and in a homozygous state in her younger sister.

## 3. Discussion

CPT II deficiency has been described as a benign condition. However, the acute crises are insidious and may occur during or after several hours of the at-risk situation and may pose a risk of death [4,8]. The disease severity may be due to (i) the associated rhabdomyolysis and its potential consequences; the nature of the infectious agent is not of importance, the energy expenditure due to this infection triggers the decompensation and the energetic imbalance leading to rhabdomyolysis (ii) the toxicity of the long chain fatty-acids and acylcarnitines on mitochondrial metabolisms [9] (iii) the decrease of free carnitine, which is necessary for long-chain fatty acid beta-oxidation notably in cardiac cells (iv) the consequences of the infectious episode.

The striking difference between the initial cause and the decompensation severity should prompt us to consider other diagnoses such as energy failure. Deciphering the symptoms linked to CPT II deficiency among those of the initial decompensation results in initiating a timely and targeted therapy. During the acute crisis, the treatment consists of glucose infusion and lipid avoidance. Long-term treatment is mainly based on an adapted diet: Avoidance of fasting, high-carbohydrate and low-fat diet, supplemented with medium chain triglycerides and carnitine. Besides, some prevention rules should be observed: Protection from infections, stress, prolonged exercise, fever, and prohibition of some medications (e.g., valproic acid). The medium-chain fatty acid triheptanoin has been described to be effective in the late-onset CPT II deficiency [1,10].

For our patient and her younger sister, medium chain triglycerides and carnitine supplementation have been initiated and to date the evolution is favorable. During the acute phase, characteristic acylcarnitine profile and clinical features guided the diagnosis and enabled the early implementation of a specific treatment. Therefore, recognition of CPT II deficiency through appropriate investigations, despite confounding factors, could be helpful to prevent dramatic consequences and subsequent triggering situations.

## Abbreviations

CPT1	Carnitine Palmitoyl transferase 1
CPT II	Carnitine Palmitoyl transferase 2
LC-MS/MS	Liquid chromatography coupled to tandem mass spectrometry
